# Kidney Specific Protein-Positive Cells Derived from Embryonic Stem Cells Reproduce Tubular Structures *In Vitro* and Differentiate into Renal Tubular Cells

**DOI:** 10.1371/journal.pone.0064843

**Published:** 2013-06-03

**Authors:** Ryuji Morizane, Toshiaki Monkawa, Shizuka Fujii, Shintaro Yamaguchi, Koichiro Homma, Yumi Matsuzaki, Hideyuki Okano, Hiroshi Itoh

**Affiliations:** 1 Department of Internal Medicine, Keio University School of Medicine, Shinjuku-ku, Tokyo, Japan; 2 Department of Physiology, Keio University School of Medicine, Shinjuku-ku, Tokyo, Japan; Wellcome Trust Centre for Stem Cell Research, United Kingdom

## Abstract

Embryonic stem cells and induced pluripotent stem cells have the ability to differentiate into various organs and tissues, and are regarded as new tools for the elucidation of disease mechanisms as well as sources for regenerative therapies. However, a method of inducing organ-specific cells from pluripotent stem cells is urgently needed. Although many scientists have been developing methods to induce various organ-specific cells from pluripotent stem cells, renal lineage cells have yet to be induced *in vitro* because of the complexity of kidney structures and the diversity of kidney-component cells. Here, we describe a method of inducing renal tubular cells from mouse embryonic stem cells via the cell purification of kidney specific protein (KSP)-positive cells using an anti-KSP antibody. The global gene expression profiles of KSP-positive cells derived from ES cells exhibited characteristics similar to those of cells in the developing kidney, and KSP-positive cells had the capacity to form tubular structures resembling renal tubular cells when grown in a 3D culture in Matrigel. Moreover, our results indicated that KSP-positive cells acquired the characteristics of each segment of renal tubular cells through tubular formation when stimulated with Wnt4. This method is an important step toward kidney disease research using pluripotent stem cells, and the development of kidney regeneration therapies.

## Introduction

Chronic kidney disease (CKD) is becoming a major global health care problem, placing a major economic strain on the health care system. Embryonic stem (ES) cells [1] and induced pluripotent stem (iPS) cells [2] have the ability to differentiate into various organs and tissues and are regarded as new tools for the elucidation of disease mechanisms as well as sources for regenerative therapies [3]. To achieve these innovative studies and therapies, however, a method of inducing organ-specific cells from pluripotent stem cells is urgently needed. In particular, renal tubular cells have not yet been successfully induced *in vitro*.

Kim et al. reported that a combination of retinoic acid, Activin, and BMP7 promoted mouse ES cells to differentiate into intermediate mesoderm and that mouse ES cells induced to differentiate using these three factors contributed to tubular epithelia when injected into mouse embryonic kidneys [4]. Similarly, Vigneau et al. showed that Activin enhanced the differentiation of mouse ES cells toward a mesoderm phenotype and that Brachyury-positive cells derived from mouse ES cells were capable of integrating into proximal tubules when injected into mouse embryonic kidneys [5]. Additionally, a number of studies tested many differentiation factors capable of promoting the differentiation of ES cells toward renal lineage cells [6]–[9].

However, a means of purification of renal tubular cells from differentiated pluripotent stem cells remains to be elucidated. Previously, we succeeded in inducing vascular cell differentiation from ES cells via the purification of Flk-1 positive cells [10]–[13], and we have also been attempting to differentiate pluripotent stem cells toward renal lineage cells. We recently found that Activin enhances the expression of kidney specific protein (KSP) in mouse ES and iPS cells [14]. KSP is a cadherin and is exclusively expressed in the kidneys. In the kidneys, KSP is expressed in the ureteric buds, developing nephrons, mesonephric tubules, Bowman’s capsules, proximal tubules, loops of Henle, distal tubules and collecting tubules [15]. Therefore, Activin is thought to enhance the differentiation of mouse ES and iPS cells toward renal tubular cells, although a method of purifying renal lineage cells from differentiated pluripotent stem cells is still needed. KSP is expressed on the cell membranes; therefore, we created a mouse monoclonal antibody against the extracellular domain of KSP and purified KSP-positive cells from differentiated mouse ES cells.

## Methods

### Culture of Undifferentiated Murine ES Cells

The mouse ES cell line, CAG-GFP EB3 was maintained as previously reported [14]. EB3 was kindly provided by Dr. Niwa (Riken Center for Developmental Biology, Kobe, Japan).

### Renal Differentiation of Mouse ES Cells and Co-culture with NIH3T3-Wnt4

To initiate the differentiation of mouse ES cells, ES cells were transferred onto 100-mm ultra low attachment culture dishes (Corning), and embryoid bodies were formed for three days. On day three of the differentiation, the embryoid bodies were transferred onto gelatin-coated cell culture dishes (Becton Dickinson) and the cells were cultured for an additional 15 days. The basal differentiation medium consisted of Dulbecco’s modified Eagle’s medium (DMEM, GIBCO), 10% FCS, and 0.1 mM 2-mercaptoethanol. Inducing factors were added separately or in combination, i.e., recombinant human/mouse/rat Activin (10 ng/mL, 338-AC; R&D Systems), recombinant mouse HGF (1, 10, 50 ng/mL, 2207-HG; R&D Systems), and recombinant mouse IGF-1 (5, 10, 100 ng/mL, 791-MG; R&D Systems).

NIH3T3-Wnt4, which was kindly provided by Dr. McMahon (Harvard Stem Cell Institute) was maintained in DMEM supplemented with 10% FCS [16]. NIH3T3-Wnt4 cells were expanded in culture dishes after being mitotically inactivated by mitomycin C (10 µg/mL) for three hours, and Matrigel (Becton Dickinson) was placed on inactivated NIH3T3-Wnt4. The KSP-positive cells were transferred onto Matrigel in Renal Epithelial Cell Growth Medium BulletKit (REGM, Lonza).

### Flow Cytometry

Mouse ES cells were collected with Trypsin-EDTA, and were subsequently treated with 0.2% collagenase type IV (GIBCO). The cells were mechanically dissociated by pipetting after incubation at 37°C for 15 min. The dissociated cells were transferred onto ultra low attachment culture dishes after replacing the medium with the basal differentiation medium, and the cells were incubated for 45 min in the basal differentiation medium at 37°C. The cells were collected into Falcon tubes and were washed with PBS twice. The cells were then treated with rat anti-mouse CD16/CD32 (Becton Dickinson) at 10 µg/mL for 20 min on ice. After that, the cells were washed with PBS containing 0.1% BSA twice, and were subsequently labeled with our anti-KSP antibody at 0.08 mg/mL for 30 min on ice. The cells were washed with PBS containing 0.1% BSA twice and were tagged with streptavidin-Alexa Fluor 647 (Invitrogen) at 0.01 mg/mL for 20 min on ice. The cells were dissociated in the basal differentiation medium supplemented with propidium iodide (Sigma), and the remaining cell aggregates were removed using a 40 µm nylon cell strainer (Becton Dickinson). The cells were analyzed and sorted using MoFlo XDP (Beckman Coulter). Cells were gated using side scatter and forward scatter, and dead cells were excluded using propidium iodide. The threshold of KSP was set according to the signal intensity of negative control samples that were incubated with no antibody and samples that were incubated with 2nd antibody (streptavidin-Alexa Fluor 647).

### RNA Extraction, cDNA Synthesis, PCR and Real-time PCR

Total RNA was isolated using RNeasy Mini kit (QIAGEN) according to the manufacturer’s instructions. Potentially contaminating genomic DNA was digested using DNase (QIAGEN). cDNA was synthesized using High-Capacity cDNA Reverse Transcription Kits (Applied Biosystems). PCR was performed with GoTaq (Promega). Real-time PCR was performed using TaqMan Fast Universal PCR Master Mix (Applied Biosystems) and Step One Plus Real-Time PCR systems (Applied Biosystems) in triplicate. The expression levels were normalized against GAPDH. Sequences of primers and probes were as follows; KSP probe- CTATCGCTGCCAGGGTGCCCAAGA, F- ATGCCCACGAAACTGTCAGC, R- TGCGTGAACACAAGAATGAGAATG; GAPDH probe- TGGTCACCAGGGCTGCCATTTGCA, F- TGAAGGTCGGTGTGAACGG, R- CCATGTAGTTGAGGTCAATGAAGG; Osr1 F- GTGACCAAGCTATCCCCAGA, R- CCAAGGGATTTTGTTGCTGT; AQP1 F- TGGCCGCAATGACCTGGCTC, R- ATGCCCAGGCCAAGCCTCCT; AQP2 F- CACATCAACCCTGCTGTGAC, R- TGTAGAGGAGGGAACCGATG; AQP3 F- TGGCTGGCCAGGTGTCTGGT, R- TGCCGGCCAGTCGTGAAGAC; Megalin F- ACTTGCTACGGGATGTGACC, R- AGGCAATGCCATCAGTAACC; Uromodulin F- TCCGTGGAGGGGACTTGCGA, R- CAGGGCACTGACCATGGGCTGTA; Slc12a3 F- CGTGGTGCCGGCCTACGAAC, R- AGGTGCCACCCGACTTGACCTT; Podocalyxin F- ATGGCGTCTACAGTGGGAAC, R- GGTCCTCATTGACCTCTGGA.

### Immunohistochemistry and Immunocytochemistry

Mouse kidney and small intestine were fixed with 4% paraformaldehyde, and were subsequently immersed in 30% (w/v) sucrose. Samples were blocked in PBS containing 5.0% (v/v) BSA after slides were made with a cryostat, and were subsequently incubated with our anti-KSP antibody, anti-AQP1 antibody (Santa Cruz), anti-Megalin antibody kindly provided by Dr. Sekine (Tokyo Metropolitan Institute of Medical Science, Japan) [17], anti-E-cadherin antibody (Abcam) and anti-AQP2 antibody (Sigma). Alexa Fluor 488 and 594 (Invitrogen) were used as a second antibody. Nuclei were stained with DAPI (Invitrogen). Pictures were taken with TCS-SP5 (Leica) and Olympus IX81.

Mouse ES cells were stained at day 18 of the differentiation with Activin (10 ng/mL) after fixation with 4% paraformaldehyde. The samples were blocked in PBS containing 5.0% BSA and were incubated with our anti-KSP antibody conjugated with biotin and anti-E-cadherin antibody (Cell Signaling). Streptoavidin-Cy5 (Beckman Coulter) and Alexa Fluor 594 (Invitrogen) were used for the detection of KSP and E-cadherin respectively.

KSP-positive and KSP-negative cells were stained on the day after the flow cytometry. The samples were treated with Endogenous Avidin/Biotin Blocking System (Abcam) and were subsequently blocked in PBS containing 5.0% BSA and 0.1% Triton. The samples were incubated with our anti-KSP antibody conjugated with biotin, anti-human specific mitochondria antibody conjugated with biotin (Abcam), anti-Megalin antibody, anti-AQP2 (Alomone Labs) or anti-Podocalyxin (R&D) antibodies. Streptavidin-Phycoerythrin (Beckman Coulter) or Alexa Fluor 594 was used for detection.

### Microarray

Total RNA was purified using RNeasy Mini kit (QIAGEN). Hybridizations were performed using Mouse Genome 430 2.0 Array (Affymetrix). The data analysis was performed using GeneSpring (Agilent). The microarray data files were deposited in the Gene Expression Omnibus (KSP-positive or -negative cells derived from mouse embryonic stem cells, GSE40694).

### Microinjection

E13.5 mouse embryonic kidneys were harvested under a stereomicroscope. KSP-positive cells derived from CAG-GFP EB3 were suspended in PBS at 1×10^8^ cells/mL after flow cytometry and were subsequently injected into mouse embryonic kidneys with Cell Tram Air 5176 (Eppendorf) using Transfer Tip (930001040, Eppendorf) and Micromanipulator 5171 (Eppendorf) under Zeiss Axiovert 135 inverted light microscope (Carl Zeiss). Approximately 10 to 20 nL of the cell suspension (1000 to 2000 cells) were microinjected beneath the renal capsules.

### Animal Experiment

Animal experiments were conducted in accordance with guidelines set by Laboratory Animal Center at Keio University School of Medicine. The protocol was approved by the Experimental Animal Committee in Keio University (Permit Number: 08090). All efforts were made to minimize suffering.

### Statistical Analysis

The results are given as the mean ± SD. A statistical analysis of the data was performed using a *t*-test or ANOVA, followed by a Tukey’s post-hoc test. P<0.05 was considered significant.

## Results

### Mouse Monoclonal Antibody Against the Extracellular Domain of KSP

**Figure 1:**
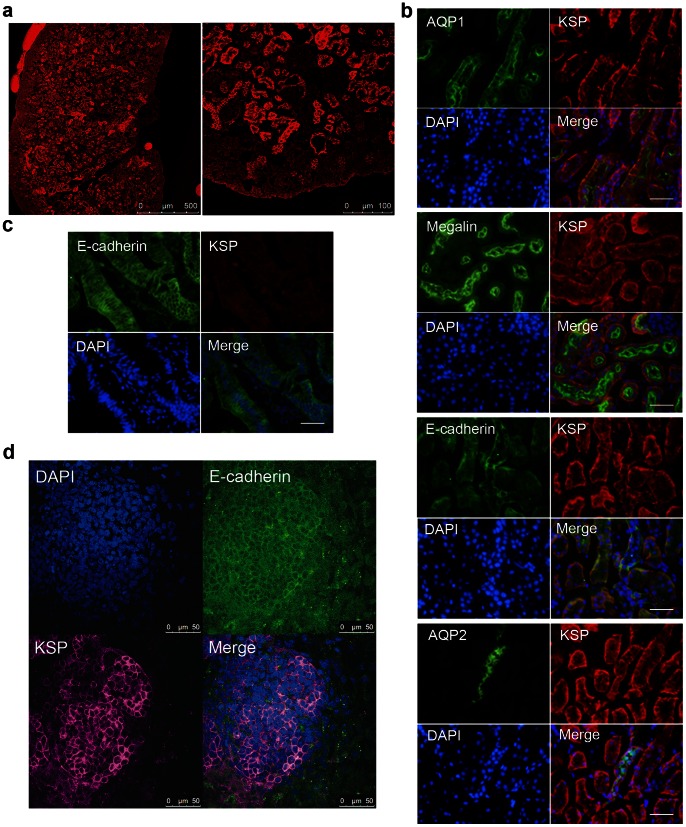
Immunohistochemistry and immunocytochemistry was performed using our original anti-KSP antibody. (a) Mouse neonate kidney stained with our anti-KSP antibody. The left picture is a low magnification, and the right picture is a high magnification. A micron scale is shown at the lower right corner. (b) Mouse kidney stained with our anti-KSP antibody, anti-AQP1 antibody, anti-Megalin antibody, anti-E-cadherin antibody and anti-AQP2 antibody. Scale bar, 400 µm. (c) Mouse small intestine stained with anti-E-cadherin antibody and our anti-KSP antibody. Scale bar, 400 µm. (d) Mouse ES cells stained using our anti-KSP antibody and anti-E-cadherin antibody after 18 days of differentiation with Activin (10 ng/mL).

The antigenicity of mouse KSP was analyzed using Epitope Adviser Lite software, and we synthesized three peptides that were expected to be located on the surface of the extracellular domain of KSP. Mice were immunized with these three peptides, and hybridomas were generated. We selected one clone that produced an antibody useful for flow cytometry after performing an evaluation using a mouse ureteric bud cell line kindly provided by Dr. Barasch (Columbia University). The antibody was conjugated with biotin to reduce the number of false-positive results. Immunofluorescent staining of mouse neonatal kidney tissue was then performed using the original anti-KSP antibody, anti-AQP1 antibody, anti-Megalin antibody, anti-E-cadherin antibody and anti-AQP2 antibody. Bowman’s capsules, the proximal to distal tubule cells, and the collecting ducts were stained by our anti-KSP antibody (Fig. 1a, b), and KSP-positive cells were co-localized with AQP1- and Megalin-positive cells which represent proximal tubules, E-cadherin-positive cells which represent distal tubules and collecting ducts, and AQP2-positive cells which represent collecting ducts. These results were consistent with those of previous reports [15], [18], [19]._ENREF_1 To test the cross-reactivity of our anti-KSP antibody to other cadherins, mouse small intestine was stained with anti-E-cadherin antibody and our anti-KSP antibody. KSP-cadherin is closely resemble liver-intestine cadherin (cadherin 17) which is abundantly expressed in small intestine [20]. However, our anti-KSP antibody did not show any positivity in small intestine, although E-cadherin was positive in small intestine (Fig. 1c).

### KSP-positive Cells Derived from Mouse ES Cells

The mouse ES cell line, EB3 [21], was differentiated through 3 days of embryoid body formation; Activin (10 ng/mL) was added to the differentiation medium throughout the differentiation period. On day 18 of the differentiation, the mouse ES cells were stained with our original anti-KSP antibody and anti-E-cadherin antibody. Our anti-KSP antibody stained the cell surface of the differentiated ES cells, and these KSP-positive cells were also weakly positive for E-cadherin (Fig. 1d). The population of KSP-positive cells was very small, and KSP-positive cells formed cell aggregations scattered along the periphery of the embryoid bodies.

**Figure 2:**
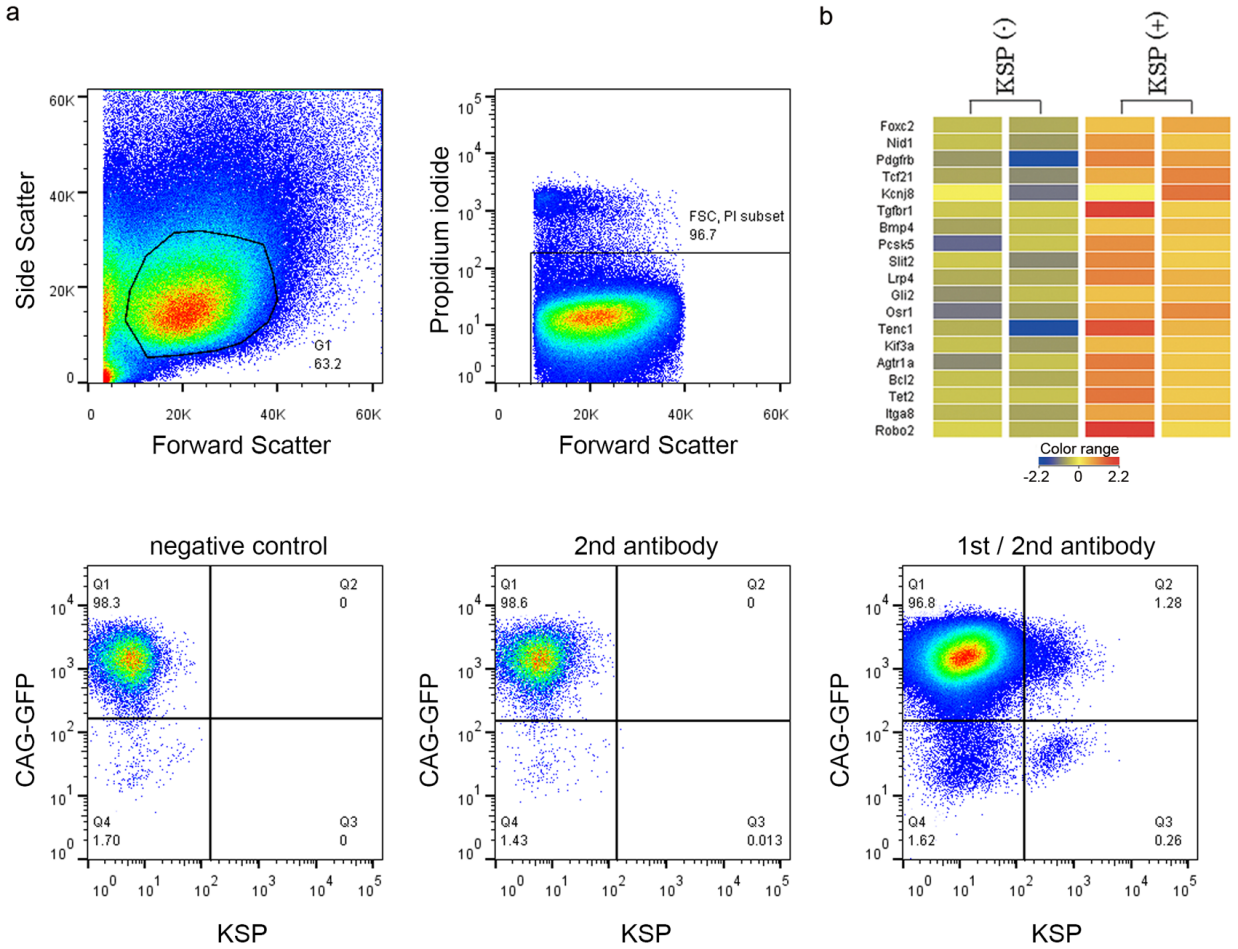
KSP-positive cells derived from mouse ES cells showed the characteristics of developing kidneys. (a) Flow cytometry for KSP in CAG-GFP EB3 on day 18 of the differentiation. Cells were gated using side scatter and forward scatter, and dead cells were excluded using propidium iodide. Negative control: Cells incubated with no antibody. 2nd antibody: Cells incubated with only streptavidin-Alexa Fluor 647. 1st/2nd antibody: Cells incubated with anti-KSP antibody and streptavidin-Alexa Fluor 647. (b) Heat map of kidney development (GO:0001822) for KSP-positive and KSP-negative cells derived from CAG-GFP EB3. The microarray data was deposited in the Gene Expression Omnibus (GSE40694, http://www.ncbi.nlm.nih.gov/geo/query/acc.cgi?acc=GSE40694).

CAG-GFP EB3 [21], which consists of EB3 ubiquitously expressing GFP (green fluorescent protein) with a CAG (cytomegalovirus immediate early gene enhancer, chicken β-actin promoter and rabbit β-globulin polyA signal) promoter was differentiated with (Activin 10 ng/mL) through 3 days embryoid body formation. On day 18 of the differentiation, the KSP-positive cells were counted using flow cytometry with our anti-KSP antibody after the exclusion of dead cells using propidium iodide (Fig. 2a). The KSP-positive cells comprised about 1% to 1.5% of the total cells. The KSP-positive and GFP-negative fraction (Fig. 2a, Q3) was considered to correspond to the dying cells, so the KSP and GFP double positive cells (Fig. 2a, Q2) and the KSP-negative and GFP positive cells (Fig. 2a, Q1) were purified using flow cytometry.

To characterize the KSP-positive cells derived from mouse ES cells, a microarray analysis of KSP-positive or KSP-negative cells purified using flow cytometry was performed. mRNAs of KSP-positive cells and KSP-negative cells day 18 of the differentiation were collected in two independent experiments. The microarray data that consist of two KSP-positive samples and two KSP-negative samples were standardized and analyzed using GeneSpring. The genes that were expressed at a level more than two-fold higher in the KSP-positive cells than in the KSP-negative cells were sorted. The genes that were up-regulated in the KSP-positive cells were grouped according to the differential expression profiles of various ontology groups, and an ontology analysis showed that genes related to kidney development (GO:0001822) (Fig. 2b) and urogenital system development (GO:0001655) were significantly up-regulated in the KSP-positive cells. On the other hand, a gene ontology analysis of the genes that were down-regulated in the KSP-positive cells showed that genes related to stem cells (GO:0048864, GO:0048863, GO:0019827) were down-regulated in the KSP-positive cells.

### HGF and IGF-1 Enhance KSP Expression Combined with Activin

**Figure 3:**
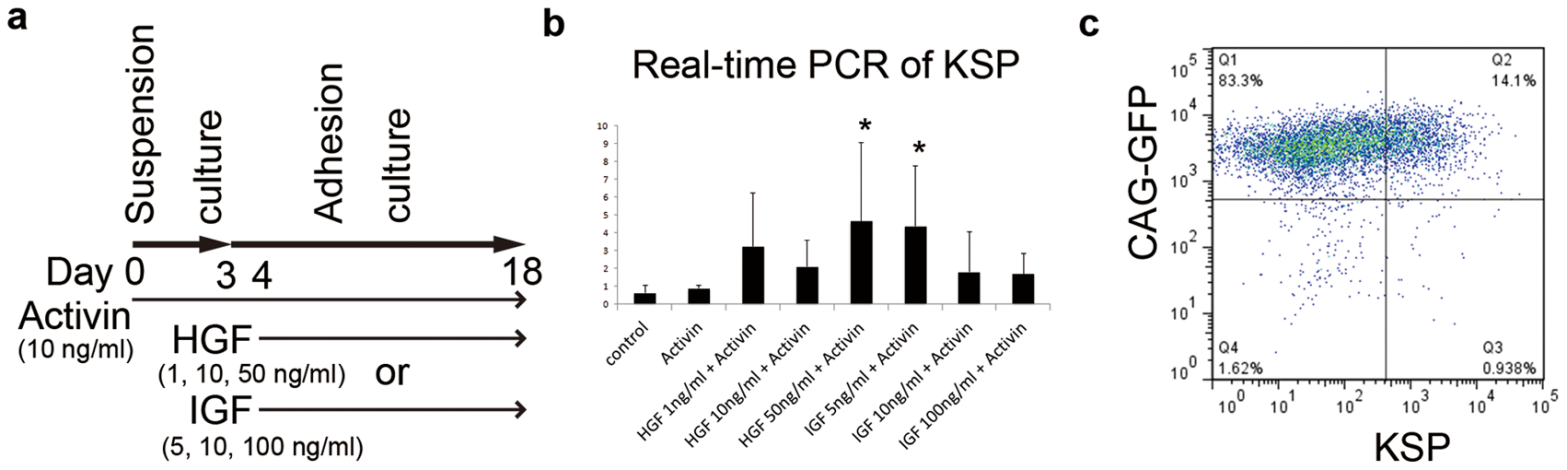
HGF and IGF-1 enhanced the expression of KSP combined with Activin. (a) Induction method for evaluating the effects of HGF and IGF-1 on mouse ES cells. (b) Expression of KSP in conditions with HGF or IGF-1 determined using real-time PCR at day 18. The values were normalized against GAPDH. The asterisk indicates *P*<0.05 versus Activin (10 ng/mL) alone. (c) Flow cytometry for KSP in CAG-GFP EB3 at day 18 of the differentiation with Activin and IGF-1.

To enhance KSP expression in mouse ES cells, from day 4 of the differentiation to day 18, hepatocyte growth factor (HGF) or insulin-like growth factor 1 (IGF-1) were added to the differentiation medium supplemented with Activin (10 ng/mL) (Fig. 3a). KSP expression was assessed using real-time PCR on day 18 of the differentiation. Both HGF and IGF-1 in combination with Activin enhanced KSP expression more than Activin alone (Fig. 3b), and only a high concentration of HGF (50 ng/mL) and a low concentration of IGF-1 (5 ng/mL) produced a statistically significant increase in KSP expression. Based on these results, we decided to add IGF-1 (5 ng/mL) to the differentiation medium from day 4 of the differentiation.

CAG-GFP EB3 cells were differentiated through three days of embryoid body formation, and Activin (10 ng/mL) was added to the differentiation medium from day 0 to day 18. In addition, IGF-1 5 ng/mL was added from day 4 to day 18. KSP-positive cells were counted using flow cytometry with our anti-KSP antibody. The percentage of KSP-positive cells ranged from 2% to 14% in more than 20 independent experiments (Fig. 3c).

### KSP-positive Cells form Tubular Structures in Matrigel

To evaluate the capacities of KSP-positive cells to form tubular structures *in vitro*, KSP-positive cells derived from CAG-GFP EB3 were transferred onto Matrigel after purification using flow cytometry. The following media were tested: DMEM +10% FBS, and REGM +10% FBS or REGM (the medium optimized for renal epithelial cell culture, and originally containing 0.5% FBS). All the media were supplemented with Activin (10 ng/mL) and IGF-1 (5 ng/mL). On the following day, the cellular morphology was observed using light microscopy.

**Figure 4:**
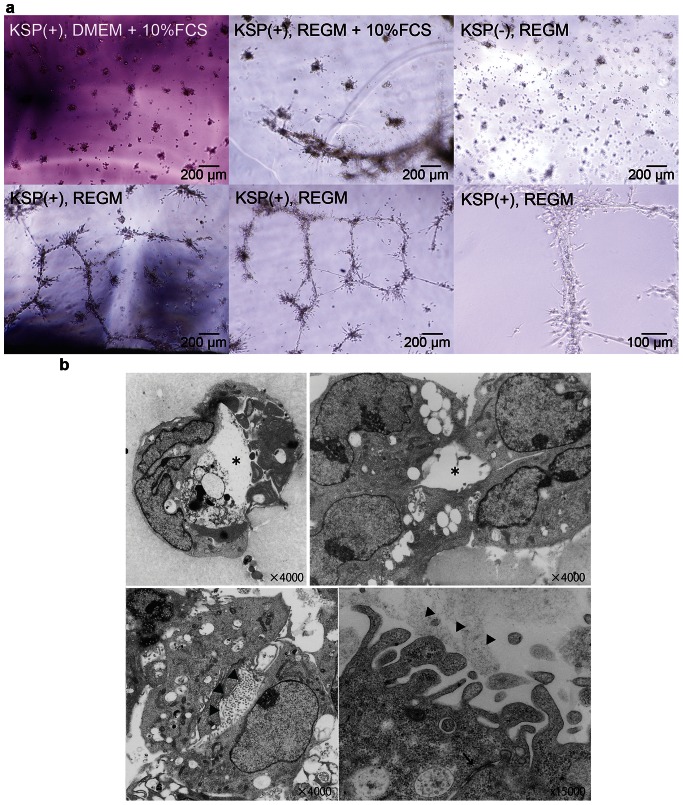
KSP-positive cells formed tubular structures in Matrigel. (a) Light microscopy of KSP-positive and KSP-negative cells under the conditions indicated in the upper left corner of the pictures on the day after flow cytometry. A micron scale is shown at the lower right corner of the pictures. (b) Electron microscopy of a cross section of the tubular structures formed by KSP-positive cells. The asterisks indicate the lumens, the arrowheads point to microvilli-like structures, and an arrow indicates a tight junction. Magnification is shown at the lower right corner.

Although KSP-positive cells did not form any tubular structures in DMEM +10% FCS and formed only a few tubular structures in REGM +10% FCS, KSP-positive cells formed abundant tubular structures in REGM alone (Fig. 4a). On the other hand, KSP-negative cells did not form any tubular structures in REGM alone.

The tubular structures were analyzed using electron microscopy after cross-section slides had been made. Luminal structures consisting of single to a few cells were observed, and microvilli-like structures were also found at the luminal surface (Fig. 4b).

However, KSP-positive cells formed these tubular structures in only two out of five independent experiments. Therefore, we tested a co-culture with NIH3T3, which ubiquitously expresses Wnt4, since Wnt4 is known to be a key auto-regulator of the mesenchymal-to-epithelial transformation and tubulogenesis [16].

### Wnt4 Promotes Tubular Formation of KSP-positive Cells

**Figure 5:**
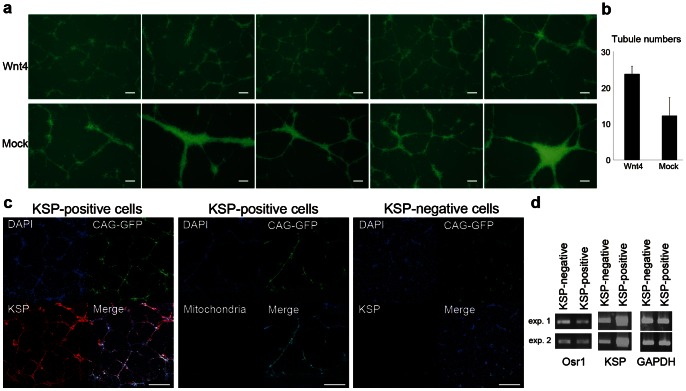
Wnt4 enhanced the tubular formation of KSP-positive cells. (a) Fluorescence microscopy of tubular structures formed by KSP-positive cells derived from CAG-GFP EB3. The upper lane shows pictures of tubular structures of KSP-positive cells co-cultured with NIH3T3-Wnt4, and the lower lane shows those co-cultured with NIH3T3-Mock. Scale bar, 200 µm. (b) Bar graph indicating the numbers of tubular structures under the indicated conditions. One tubular structure from a branching to a branching is counted as one. (c) Immunofluorescence of KSP-positive or KSP-negative cells showing KSP and human-specific mitochondria as a negative control. Scale bar, 400 µm. (d) PCR showing the expression of Osr1, KSP and GAPDH of KSP-positive and -negative cells after three days of 3D culture in Matrigel with NIH3T3-Wnt4.

After cell purification using our anti-KSP antibody, KSP-positive cells were transferred onto Matrigel which was directly placed on NIH3T3-Wnt4 cells treated with mitomycin C, then cultured for 24 hours. Co-culture with NIH3T3-Wnt4 significantly promoted tubular formation and the branching of KSP-positive cells, compared with co-culture with NIH3T3-Mock cells (Fig. 5a). The numbers of branched tubular structures were counted at the center of the dishes (n = 5) and were significantly greater with Wnt4 than with Mock (Fig. 5b). The KSP-negative cells were also co-cultured with NIH3T3-Wnt4; however, the KSP-negative cells did not produce any tubular structures in more than 10 independent experiments.

These tubular structures were stained using our anti-KSP antibody conjugated with biotin or human-specific anti-mitochondria antibody conjugated with biotin as a negative control (Fig. 5c). The tubular structures that were formed by KSP-positive cells were positive for KSP; however, the KSP-negative cells were barely positive for KSP.

PCR was also performed to compare the gene expressions of KSP-positive and KSP-negative cells after three days of culture in Matrigel with NIH3T3-Wnt4. KSP was highly expressed in the KSP-positive cells, though a low level of KSP expression was also observed in the KSP-negative cells under Wnt4 stimulation. On the other hand, the expression of Osr1 was down-regulated in the KSP-positive cells through tubular formation (Fig. 5d). In light of the microarray results for KSP-positive and KSP-negative cells right after cell purification, KSP-positive cells were thought to have differentiated toward epithelial cells and to have lost the characteristics of metanephric mesenchymal cells through tubular formation.

### KSP-positive Cells Differentiate into Renal Epithelial Cells through Tubular Formation and Incorporate with Renal Tubular Cells of Mouse Embryonic Kidney

**Figure 6:**
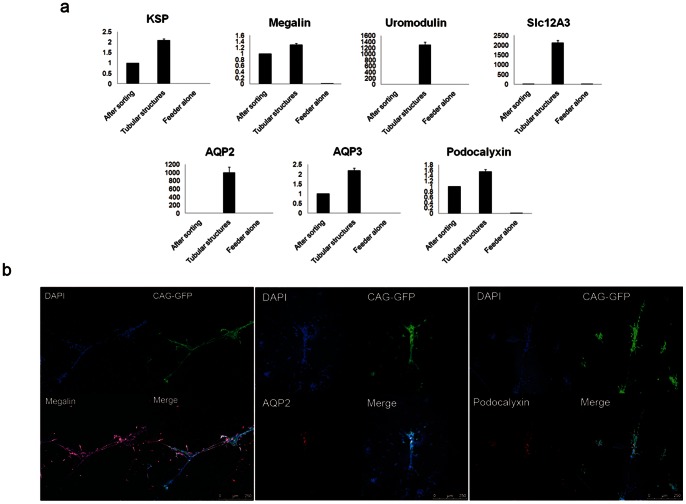
Differentiation of KSP-positive cells into each segment of renal tubular cells. (a) PCR showing the expression of segment-specific genes of renal tubular cells. The samples were KSP-positive cells examined right after cell purification with flow cytometry, KSP-positive cells forming tubular structures co-cultured with NIH3T3-Wnt4, and NIH3T3-Wnt4 alone. The bands were quantified using ImageJ, and graphs normalized against GAPDH were shown. After sorting: a sample of cells collected right after cell sorting of KSP-positive cells. Tubular structures: a sample of KSP-positive cells that form tubular structures and feeder cells of NIH3T3-Wnt4. Feeder alone: a sample of feeder cells, NIH3T3-Wnt4. (b) Immunofluorescence showing Megalin, AQP2 and Podocalyxin in tubular structures formed by KSP-positive cells co-cultured with NIH3T3-Wnt4. A micron scale is shown at the lower right corner.

The tubular structures formed by KSP-positive cells under Wnt4 stimulation were characterized using PCR and immunocytochemistry. PCR showed that KSP-positive cells expressed Megalin, Uromodulin, Slc12A3, AQP2, AQP3 and Podocalyxin after tubular formation under the stimulation of Wnt4 (Fig. 6a). Immunocytochemistry also revealed that these tubular structures were positive for Megalin, AQP2 and Podocalyxin (Fig. 6b). These results suggested that KSP-positive cells have the capacity to differentiate into each segment of renal tubular cells.

The differentiation capacities of KSP-positive cells were also confirmed using transplantation experiments. E13.5 mouse fetuses were extracted from pregnant mice under anesthesia, and embryonic kidneys were harvested from the abdominal cavities of the mouse fetuses. KSP-positive cells were purified using flow cytometry and were injected into the mouse embryonic kidneys under a microscope with a manual microinjector. The mouse embryonic kidneys were then cultured in the upper chambers of a transwell for 3 days with NIH3T3-Wnt4 co-cultured in the lower chambers.

**Figure 7:**
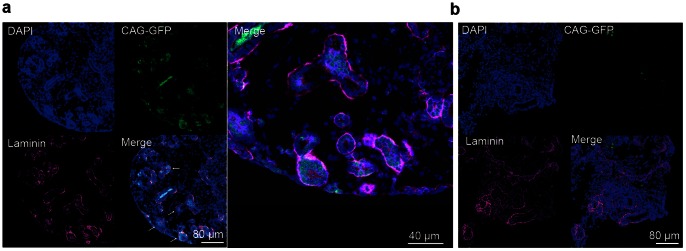
KSP-positive cells were incorporated with renal tubular cells of mouse embryonic kidneys. KSP-positive and KSP-negative cells were injected into mouse embryonic kidneys. An immunofluorescent analysis was performed on day 3 after the injection. A micron scale is shown at the lower right corner. (a) KSP-positive cells. The arrows indicate the ES cells that incorporated with the renal tubular cells, and a high magnification picture is also shown. (b) KSP-negative cells.

Frozen sections were made on day 3 of the organ culture. The basement membranes were stained with anti-laminin antibody. An immunofluorescent study revealed that KSP-positive cells derived from mouse ES cells were incorporated with renal tubular cells (Fig. 7a). KSP-negative cells were also injected into mouse embryonic kidneys; however, KSP-negative cells failed to proliferate and to be incorporated with renal tubular cells (Fig. 7b).

## Discussion

We succeeded in purifying KSP-positive cells from mouse ES cells via a differentiation culture with Activin; however, the percentage of KSP-positive cells was too low to obtain a sufficient amount of cells required for further experiments. Thus, we looked for other inducing factors capable of promoting the expression of KSP in mouse ES cells and tested the effects of HGF and IGF-1. Woolf et al. reported that HGF is required for the differentiation of metanephric mesenchymal cells into epithelial precursors [22]. On the other hand, Rogers et al. showed that IGF-1 is produced by metanephroi and is essential for kidney development [23]. Moreover, Hammerman et al. reported that IGF-1 or HGF accelerates the restoration of kidney function and the normalization of histology after acute renal injury, reducing mortality [24]. Our results were compatible with these previous reports and showed that both HGF and IGF-1 could enhance the expression of KSP in mouse ES cells; consequently, we decided to use a low concentration of IGF-1 combined with Activin. Using a combination of IGF-1 and Activin, we were able to induce the differentiation of mouse ES cells into KSP-positive cells more efficiently.

Based on microarray results, we thought that KSP-positive cells purified from differentiated mouse ES cells exhibited the characteristics of metanephric mesenchyme, and PCR revealed that KSP-positive cells lacked tubular segment specific genes (Fig. 6a). In other words, KSP-positive cells seem to represent progenitor-like cells of renal tubular cells. Therefore, we needed to induce the differentiation of KSP-positive cells toward renal epithelial cells, and we suspected that tubular formation might be vital for this step. The *in vitro* reproduction of nephron structures is a challenging issue; however, Taub et al. succeeded in the formation of tubular structures from primary baby mouse kidney epithelial cell cultures using Matrigel. Taub et al. showed electron microscopy pictures indicating luminal formation and microvilli structures at the luminal surface [25]. However, the structures lacked basement membranes, and nephrons are still difficult to reproduce *in vitro*.

To promote such tubular formation, we tested the effects of Wnt4 which is known to be required for tubular formation [16], [26]. Our experiments showed that co-culturing with NIH3T3-Wnt4 promoted the tubular formation of KSP-positive cells. These cells formed tubular structures that expressed the segment-specific genes of renal tubular cells, i.e., Megalin expressed in proximal tubules, Uromodulin expressed in loops of Henle, Slc12A3 expressed in distal tubules, AQP2 and AQP3 expressed in collecting ducts, and Podocalyxin expressed in Bowman’s capsules and podocytes [27]–[30]. We also performed a regular adhesion culture after cell purification using flow cytometry; however, PCR showed no expression of Uromodulin, Slc12A3, and AQP2 even after stimulation with Wnt4 using the supernatant of NIH3T3-Wnt4 cell cultures (data not shown). These results indicated that 3D extracellular matrix is essential for KSP-positive cells to form tubular structures and differentiate into matured renal tubular cells, and further experiments are required to examine what extracellular matrix such as collagen or laminin is required for the tubular formation and the differentiation of renal tubular cells.

Our results indicated that KSP-positive cells acquired the characteristics of each segment of renal tubular cells through tubular formation. Based on a microarray analysis of KSP-positive cells, we thought the KSP-positive cells had the characteristics of immature renal tubular cells and could be differentiated toward renal tubular cells through tubular formation.

In conclusion, we induced renal tubular cells from mouse ES cells via the cell purification of KSP-positive cells. Further experiments are still necessary to establish the segment-specific induction of tubular cells and podocytes; however, our method will contribute to disease-specific iPS research on kidneys and the development of renal regeneration therapies.
